# The Prognostic Value of Harvested Lymph Nodes and the Metastatic Lymph Node Ratio for Gastric Cancer Patients: Results of a Study of 1,101 Patients

**DOI:** 10.1371/journal.pone.0049424

**Published:** 2012-11-15

**Authors:** Shi Chen, Bai-Wei Zhao, Yuan-Fang Li, Xing-Yu Feng, Xiao-Wei Sun, Wei Li, Zhi-Wei Zhou, You-Qing Zhan, Chao-Nan Qian, Ying-Bo Chen

**Affiliations:** 1 Department of Gastropancreatic Surgery, Sun Yat-sen University Cancer Center, Guangzhou, Guangdong, P. R. China; 2 State Key Laboratory of Oncology in South China, Sun Yat-sen University Cancer Center, Guangzhou, Guangdong, P. R. China; 3 Laboratory of Cancer and Developmental Cell Biology, Van Andel Research Institute, Grand Rapids, Michigan, United States of America; Virginia Commonwealth University School of Medicine, United States of America

## Abstract

**Aim:**

To investigate whether the recommendation to remove 15 lymph nodes that is used in the staging system is necessary to assess gastric cancer progression and to evaluate whether our metastatic lymph node ratio dividing method, adapted from the AJCC’s (American Joint Committee on Cancer) 7^th^ TNM staging system, is helpful for the patients with fewer than 15 harvested lymph nodes.

**Methods:**

We performed a retrospective study of 1101 patients with histologically diagnosed gastric cancer who underwent a D2 gastrectomy at the Sun Yat-sen University Cancer Center between January 2001 and December 2010. The Kappa and Chi-squared tests were employed to compare the clinicopathological variables. The Kaplan-Meier method and Cox regression were employed for the univariate and multivariate survival analyses.

**Results:**

In the trial, 346, 601 and 154 patients had 0–14, 15–30 and more than 30 lymph nodes harvested, respectively. The median survival times of patients with different lymph nodes harvested in N0, N1, N2 and N3a groups were 45.43, 54.28 and 66.95 months (p = 0.068); 49.22, 44.25 and 56.72 months (p<0.001), 43.94, 47.97 and 35.19 months (p = 0.042); 32.88, 42.76 and 23.50 months (p = 0.016). Dividing the patients who had fewer than 15 lymph nodes harvested by the metastatic lymph node ratio at 0, 0.13 and 0.40, the median survival times of these 4 groups were 70.6, 50.5, 53.5 and 30.7 months (p<0.001). After re-categorising these 4 groups into the N0, N1, N2, N3a groups, the histological grade, T staging, premier N staging, and restaged N staging were the independent prognostic factors.

**Conclusions:**

Large numbers of lymph nodes harvested in radical gastrectomy do not cause stage migration. For those patients with a small number of harvested lymph nodes, their stage should be divided by the metastatic lymph node ratio, referred to in the TNM staging system, to assign them an accurate stage.

## Introduction

Approximately one million people are diagnosed with gastric cancer each year, making it the fourth most common cancer type and the second leading cause of cancer-related death worldwide, with an estimated 800,000 deaths caused by the disease [1]. More new cases are diagnosed in China than in other countries around the world [2], and most of those patients are diagnosed at an advanced disease stage [3], [4]. Surgery is the only way to cure gastric cancer in these patients. Gastric resection may be classified by the extent of lymph node dissection at surgery. A D2 radical gastrectomy is considered a standard surgical procedure in Asian countries especially Japan, South Korea and China, although Western investigators have not found a survival advantage when extensive lymphadenectomy is compared with a D1 resection [5]–[8]. The prognosis for gastric cancer patients undergoing a D2 resection remains very poor, which may be due to the inaccurate post-surgical staging for patients and a subsequent inappropriate choice of adjuvant treatment.

In 2010, the AJCC’s (American Joint Committee on Cancer) 7th edition TNM classification of malignant tumours for gastric cancer was published [9]. Primary tumours (T), regional lymph nodes (N) and metastasis (M) are the three most important independent prognostic factors for gastric cancer patients. Among these factors, the regional lymph nodes are the most difficult to accurately stage. The number of lymph nodes to be removed in surgery is not clearly defined in a D2 resection. The resection of 15 nodes is recommended in the AJCC’s TNM staging system. Another question is whether more accurate staging can be achieved by removing more lymph nodes. Schwarz et al. [10] found that a stage-based survival prediction depends on the total lymph node number and the number of negative lymph nodes. Other investigators have suggested that 20, or even 30, lymph nodes is a better choice than 15 [11], [12]. Some investigators suggest the use of the metastatic lymph node ratio to eliminate the variability generated by removing different numbers of lymph nodes. Additionally, they found that the metastatic lymph node ratio is an independent prognostic factor [13]–[15]. The metastatic lymph node ratio has not previously been accepted as a standard for staging gastric cancer. The reasons for this lack of acceptance may be that different investigators have used a variety of dividing methods to determine the metastatic lymph node ratios, and stronger evidence is required to support the metastatic lymph node ratio as a standard for determining the N portion of the TNM staging system for gastric cancer as a replacement for the current standard of assessing the regional lymph node numbers.

In our study, we investigated whether recommended 15 lymph nodes for use in the TMN staging system is sufficient for evaluating gastric cancer and whether 30 lymph nodes would be more accurate. Additionally, we assessed whether our metastatic lymph node ratio dividing method, adapted from the AJCC’s 7^th^ TNM staging system, is helpful for the patients with fewer than 15 harvested lymph nodes.

## Materials and Methods

### Ethics Statement

All patients were provided written informed consent for their information to be stored in the hospital database. Study approval was obtained from the independent ethics committees at Cancer Center of Sun Yat-Sen University. The study was undertaken in accordance with the ethical standards of the World Medical Association Declaration of Helsinki.

### Eligibility Criteria

The eligibility requirements included the following: (1) Patients had gastric carcinoma identified by histopathological examination, (2) underwent gastrectomy, (3) presented absence of identifiable distant metastasis, such as liver, lung and distal lymph nodes, (4) presented no history of another synchronous malignancy, (5) presented no recurrent gastric cancer or remnants of gastric cancer, (6) received no neoadjuvant therapy, (7) survived in the perioperative period and (7) had complete follow-up data collection. The procedures of tumour resection and the D2 lymphadenectomy performed by experienced surgeons were similar in all patients undergoing radical resection.

### Surgical Procedures

**Figure 1:**
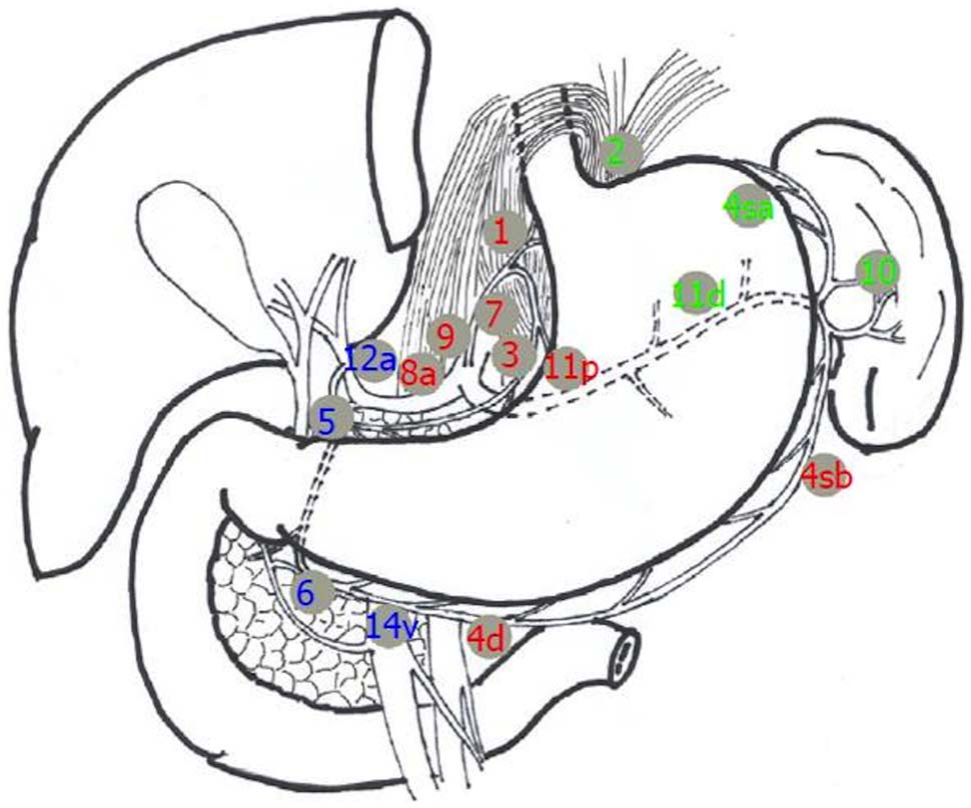
The type of gastrectomy and the extent of the D2 dissection were determined by the tumour location.

According to the guidelines of the Japanese Gastric Cancer Association (JGCA), the stomach was divided anatomically into upper, middle and lower portions. The three portions were defined by subdividing both the lesser and greater curvatures into three equal lengths [16]. The type of gastrectomy and extent of the D2 dissection were determined by the tumour location (Figure 1) [16]. The aim of any oncological resection was to achieve en-bloc resection of the gastric segment and surrounding lymph nodes to obtain adequate oncological clearance. Forty-eight patients received a concurrent splenectomy as part of their surgical treatment, and 23 of these also underwent a resection of the pancreatic body and tail.

#### Method for recategorising N staging

It is well known that in the AJCC’s TNM staging system, the cut-off values to divide the patients into different N groups are 0, 2 and 6 regional lymph nodes. Thus, we evaluated the N staging of patients by the metastatic lymph node ratio with 0, 0.13 (2/15), and 0.40 (6/15) as our cut-off values. According to the metastatic lymph node ratio, we separated the patients who had fewer than 15 lymph nodes harvested into 4 groups [0, 0–0.13 (2/15), 0.13–0.40 (6/15) and greater than 0.40] and re-categorised these 4 groups into the N0, N1, N2, and N3a groups. These groups were then combined with other patients who had greater than 15 lymph nodes harvested to create a revised N staging.

### Patient Characteristics

We included 1,101 patients who underwent gastrectomy at the Sun Yat-sen University Cancer Center between January 2001 and December 2010. The postoperative pathological results include tumour size, histological type, margin, adjacent tissues and neighbouring organs, retrieved lymph nodes, metastatic lymph nodes, and pTNM staging. The eligibility criteria include histologically confirmed R0 resection, which was defined as no residual macroscopic or microscopic tumour. Patients with distant metastases or carcinoma of the gastric stump after a gastric resection for benign disease were excluded from the study.

### Patient Follow-up

After treatment, patients with advanced gastric cancer were monitored every 2–3 weeks for six months postoperatively and then every 3 months for the first 2 years. Patients with early stage gastric cancer were required to have a further consultation with the doctor every 3 months for the first 2 years. All of the patients were monitored every 6 months thereafter. Telephone calls and letters were used to assess patients who could not be physically present for follow-up. Complete data were collected from all 1101 patients from the time following treatment until July 2011. The follow-up period ranged from 6 to 120 months (median, 41 months).

### Statistical Analysis

The Kappa and Chi-squared tests were used to compare the clinicopathological variables between the groups with different numbers of harvested lymph nodes. Univariate survival analysis was performed using the Kaplan-Meier method. Survival curves were compared with the log-rank test. Multivariate statistical survival analysis was performed using the Cox regression. Analyses were performed with the SPSS software version 20.0 for Windows (SPSS, Inc., Chicago, IL). Statistical significance was defined as P<0.05.

## Results

### Patient Demographics

In total, 2 patients died in the perioperative period, secondary to anastomotic leakage and renal failure. There were another 94 patients excluded from our study because of incomplete follow-up data. The total number of patients included in our study is 1101. The median age of them was 59 years old (range: 18–83). Of these patients, 752 were male and 349 were female. The 5-year survival of the whole patient group was 41.0%, with a median survival of 61.2 months. The patient clinicopathological characteristics are presented in Table 1. The average number of lymph nodes harvested was 21.51±12.42 (mean ± standard deviation) and a median of 20 (range: 1–68). The average number of metastatic lymph nodes harvested was 4.26±6.04 (range:0–56). According to the 7^th^ AJCC’s TNM staging system for gastric cancer, there were 351, 219, 224, 233 and 74 patients in the N0, N1, N2, N3a and N3b groups, respectively. There were 93, 65, 41, 773 and 129 patients in the T0, T1, T2, T3, T4a and T4b groups, respectively. The clinicopathological factors for all 1101 patients are presented in Table 1.

**Table 1:** Clinical pathological data for gastric cancer patients with different numbers of harvested lymph nodes.

Clinical pathological data		Fewer than 15 harvested lymph nodes (n = 346 cases)		Harvested lymph nodes (15–29) (n = 601 cases)			Greater than 30 harvested lymph nodes (n = 154 cases)			
		Cases	%	Cases		%	Cases		%	P value
Age (Years)	Median	61		57			58			0.316
	Range	28–83		18–82			22–76			
Sex	Male	245	70.8	401	66.7		106	68.8		
	Female	101	29.2	200	33.3		48	31.2		0.424
Tumour location	Gastric cardia	211	61.0	232	38.6		42	27.3		
	Middle	29	8.4	115	19.1		31	20.1		
	Antrum	84	24.3	234	38.9		75	48.7		
	Total stomach	2	0.6	7	1.2		4	2.6		
	Remnant stomach*	20	5.8	13	2.2		2	1.3		<0.001
Surgery	Proximal gastrectomy	218	63.0	219	36.4		42	27.3		
	Distal gastrectomy	93	26.9	293	48.8		85	55.2		
	Total gastrectomy	35	10.1	89	14.8		27	17.5		<0.001
Tumour size	<3 cm	52	15.0	101	16.8		14	9.1		
	≥3 cm	294	85.0	500	83.2		140	90.9		0.059
Borrmann type	I	12	3.8	18	3.4		2	1.3		
	II	184	57.5	286	53.4		60	39.5		
	III	107	33.4	210	39.2		77	50.7		
	IV	17	5.3	22	4.1		13	8.6		0.002
Histological grade	High-differentiation	4	1.2	6	1.0		0	0		
	Median-differentiation	111	32.1	147	24.5		23	14.9		
	Low-differentiation	164	47.4	330	54.9		102	66.2		
	Poor-differentiation**	67	19.4	118	19.6		29	18.8		0.001
T staging***	T1	26	7.5	65	10.8		2	1.3		
	T2	16	4.6	45	7.5		4	2.6		
	T3	9	2.6	32	5.3		0	0.0		
	T4a	233	67.3	348	57.9		133	86.4		
	T4b	62	17.9	111	18.5		15	9.7		<0.001
N staging***	N0	133	38.4	185	30.8		33	21.4		
	N1	79	22.8	119	19.8		21	13.6		
	N2	94	27.2	87	14.5		43	27.9		
	N3a	40	11.6	156	26.0		37	24.0		
	N3b	0	0.0	54	9.0		20	13.0		<0.001
Adjuvant Chemotherapy	Yes	254	73.4	442	73.5		121	78.6		
	No	92	26.6	159	26.5		33	21.4		0.410

* These 35 remnant stomach patients all had a more than 5 years history of the gastrectomy because of the gastric ulcer.

** Poorly-differentiated cells: signet ring cell carcinoma, mucinous adenocarcinoma, undifferentiated carcinoma, etc.

*** The T and N stagings for this group of patients are defined according to the AJCC’s 7^th^ TNM staging system for gastric cancer.

### Significance of the Differing Numbers of Harvested Lymph Nodes on the Prognosis of Different N Staging

**Figure 2:**
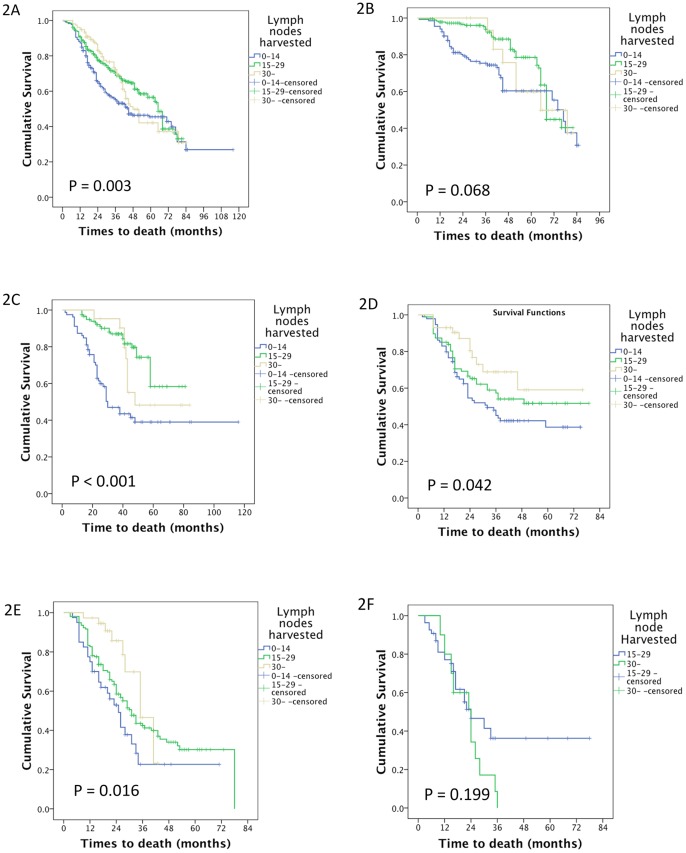
The survival of patients with different numbers of harvested lymph nodes. A The median survival times of patients who had 0–14, 15–30 and more than 30 lymph nodes harvested in the study were 44.94, 46.62 and 42.82 months, respectively (p = 0.003). B The median survival times of patients who had 0–14, 15–30 and more than 30 lymph nodes harvested in the N0 group were 45.43, 54.28 and 66.95 months, respectively (p = 0.068). C The median survival times of patients who had 0–14, 15–30 and more than 30 lymph nodes harvested in the N1 group were 49.22, 44.25 and 56.72 months, respectively (p<0.001). D The median survival times of patients who had 0–14, 15–30 and more than 30 lymph nodes harvested in the N2 group were 43.94, 47.97 and 35.19 months, respectively (p = 0.042). E The median survival times of patients who had 0–14, 15–30 and more than 30 lymph nodes harvested in the N3a group were 32.88, 42.76 and 23.50 months, respectively (p = 0.016). F The median survival times of patients who had 15–30 and more than 30 lymph nodes harvested in the N3b group were 22.48 and 36.0 months, respectively (p = 0.199).

There were 346, 601 and 154 patients who had 0–14, 15–30 and more than 30 lymph nodes harvested, respectively. Their median survival times were 44.94, 46.62 and 42.82 months, respectively (p = 0.003). The results are shown in figure 2A.

In the N0 group, there were 133, 186 and 32 patients with 0–14, 15–30 and more than 30 lymph nodes harvested, respectively. Their median survival times were 45.43, 54.28 and 66.95 months, respectively (p = 0.068). These results are shown in figure 2B.

In the N1 group, there were 79, 119 and 21 patients with 0–14, 15–30 and more than 30 lymph nodes harvested, respectively. Their median survival times were 49.22, 44.25 and 56.72 months, respectively (p<0.001). These results are shown in figure 2C.

In the N2 group, there were 94, 87 and 43 patients with 0–14, 15–30 and more than 30 lymph nodes harvested, respectively. Their median survival times were 43.94, 47.97 and 35.19 months, respectively (p = 0.042). These results are shown in figure 2D.

In the N3a group, there were 40, 156 and 37 patients with 0–14, 15–30 and more than 30 lymph nodes harvested, respectively. Their median survival times were 32.88, 42.76 and 23.50 months, respectively (p = 0.016). These results are shown in figure 2E.

In the N3b group, there were 53 and 21 patients with 15–30 and more than 30 lymph nodes harvested, respectively. Their median survival times were 22.48 and 36.0 months, respectively (p = 0.199). The results are shown in figure 2F.

### Significance of the Metastatic Lymph Node Ratio on the Prognosis of Patients with Different N Stagings who had Fewer than 15 Lymph Nodes Harvested

**Figure 3:**
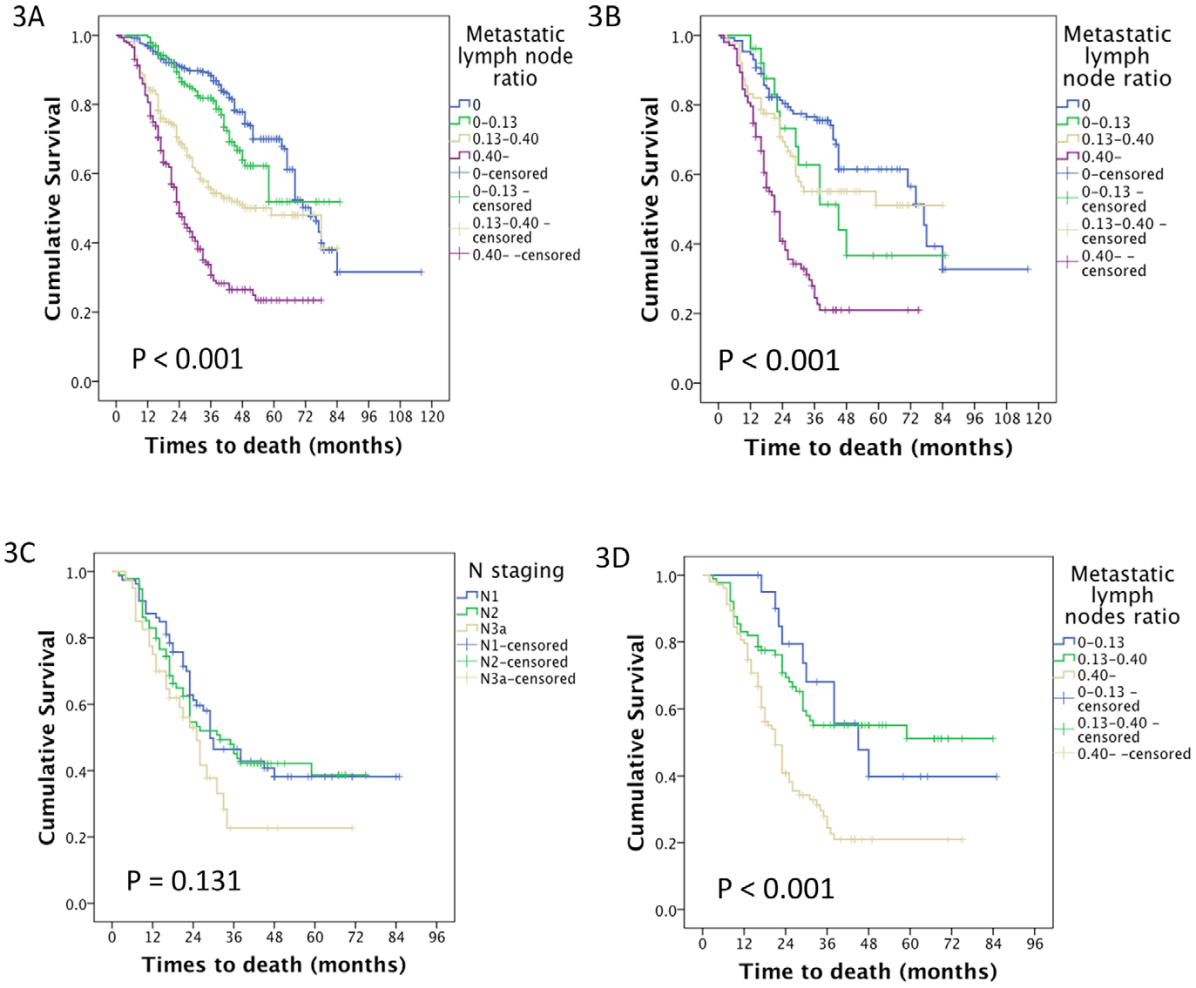
The survival of patients with different metastatic lymph nodes ratio. A The median survival times of patients who had metastatic lymph node ratios of 0, 0–0.13 (2/15), 0.13–0.40 (6/15) and more than 0.40 in the study were 75.0, 62.5, 51.4 and 31.6 months, respectively (p<0.001). B The median survival times of patients who had metastatic lymph node ratios of 0, 0–0.13 (2/15), 0.13–0.40 (6/15) and more than 0.40 in the group with fewer than 15 harvested lymph nodes group were 70.6, 50.5, 53.5 and 30.7 months, respectively (p<0.001). C The median survival times of patients in the N1, N2 and N3a groups, those patients who had at least one metastatic lymph node in the fewer than 15 nodes that were harvested, were 46.2, 41.8 and 30.9 months, respectively (p = 0.131). D The median survival times of patients whose metastatic lymph node ratios were 0, 0–0.13 (2/15), 0.13–0.40 (6/15) and more than 0.40 in the group who had at least one metastatic lymph node in the fewer than 15 nodes that were harvested were 53.5, 53.5 and 30.7 months, respectively (p<0.001).

We categorised the patients in this study by their metastatic lymph node ratios: 0, 0–0.13 (2/15), 0.13–0.40 (6/15) and greater than 0.40; there were 351, 195, 270 and 285 patients in these 4 groups, respectively. Their median survival times were 75.0, 62.5, 51.4 and 31.6 months, respectively (p<0.001). The survival curve is shown in Figure 3A. For the patients with fewer than 15 harvested lymph nodes, there were 133, 21, 89 and 103 patients in the 4 groups, respectively. Their median survival times of these 4 groups were 70.6, 50.5, 53.5 and 30.7 months, respectively (p<0.001). The survival curve is shown in Figure 3B.

We focused on the patients who had fewer than 15 lymph nodes harvested and were found to have at least one metastatic lymph node. There were 213 patients in this category. According to the AJCC’s TNM staging system, there were 79, 94 and 40 patients in N1, N2 and N3a groups, respectively; their median survival times were 46.2, 41.8 and 30.9 months, respectively (p = 0.131). A survival curve is shown in Figure 3C. According to the metastatic lymph node ratio, there were 21, 89 and 103 patients in each group; their median survival times were 53.5, 53.5 and 30.7 months, respectively (p<0.001). A survival curve is shown in Figure 3D.

### Univariate and Multivariate Analyses for Restaging the N Staging of these Gastric Cancer Patients

**Figure 4:**
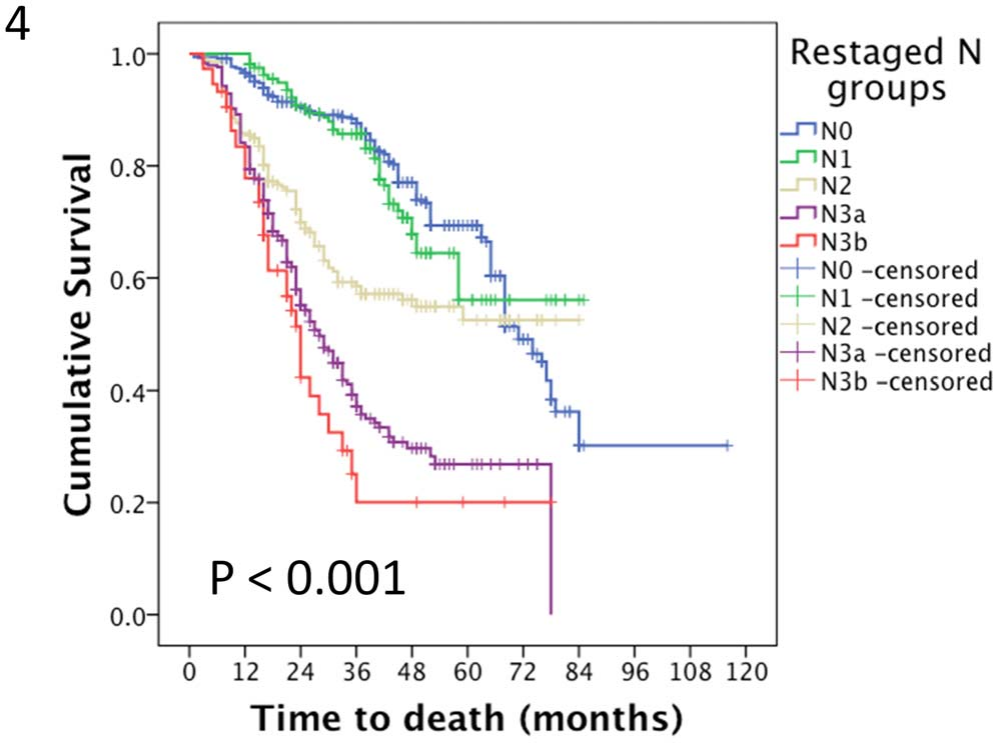
The median survival times of patients in the re-categorised N1, N2, N3a and N3b groups patients were 74.6, 64.9, 54.5 37.7 and 31.6 months, respectively (p<0.001).

According to the metastatic lymph node ratio, we separated the patients who had fewer than 15 lymph nodes harvested into 4 groups [0, 0–0.13 (2/15), 0.13–0.40 (6/15) and more than 0.40], and re-categorised these patients into the N0, N1, N2 and N3a groups. There were 351, 161, 219, 296 and 74 patients in the N0, N1, N2, N3a and N3b groups, respectively. Their median survival times were 74.6, 64.9, 54.5 37.7 and 31.6 months, respectively (p<0.001). A survival curve is shown in Figure 4.

In the univariate analysis, the tumour size, tumour position, histological grade, Borrmann type, T staging, premier N staging, and restaged N staging significantly correlated with the overall survival (Table 2). In the multivariate analysis, the number of lymph nodes harvested, T staging and premier N staging were independent factors (Table 3). When we included the restaged N staging in the Cox’s regression model, the histological grade, T staging, premier N staging and restaged N staging were independent factors (Table 4). The hazard ratio (HR) value of the restaged N staging system was higher than that of the premier N staging.

**Table 2:** Univariate analysis of the overall survival in local, late-stage gastric cancer patients.

Variables	n	5-year survival rate %	Median survival (months)	P value
**All 1101 gastric cancer patients**				
**Tumour location**				<0.001
Gastric cardia	485	40	60.1	
Middle	175	27	48.6	
Antrum	393	51	60.3	
Total stomach	13	32	41.6	
Remnant stomach	35	0	37.4	
**Tumour size**				<0.001
<3 cm	167	64	67.4	
≥3 cm	934	37	58.9	
**Borrmann type**				<0.001
I	32	44	53.4	
II	530	38	63.6	
III	394	36	49.8	
IV	52	18	32.4	
**Histological grad**e				0.007
High-differentiation	10	90	76.4	
Median-differentiation	281	50	58.4	
Low-differentiation	596	36	60.9	
Poor-differentiation	214	33	52.4	
**T staging**				<0.001
T1	93	93	80.1	
T2	65	77	65.9	
T3	41	77	70.3	
T4a	714	35	58.9	
T4b	188	21	39.6	
**N staging**				<0.001
N0	351	49	64.5	
N1	219	53	75.7	
N2	224	47	48.5	
N3a	233	29	39.9	
N3b	74	19	31.5	
**Lymph nodes harvested**				0.003
0–14	346	33	44.1	
15–29	601	38	64.0	
30–	154	38	50.6	
**Restaged N staging**				<0.001
N0	351	49	74.6	
N1	161	57	64.9	
N2	219	53	54.5	
N3a	296	27	37.7	
N3b	74	19	31.6	

## Discussion

Lymph node metastasis is one of the most important prognostic factors in gastric cancer. The AJCC’s 7^th^ TNM staging system for gastric cancer is commonly used in the clinic. However, this system recommends the dissection of more than 15 lymph nodes for N staging, except for the N0 patients. It is unclear whether the 15 nodes can be a standard for the D2 radical gastrectomy, or whether 30 would be better. Another argument is the loose definition of the D2 dissection [17]. A recommended number of lymph nodes to dissect intraoperatively may help standardise the surgical procedure. Patients may have fewer than 15 lymph nodes harvested for many reasons. Two main reasons are that the number of lymph nodes harvested is dependent both on surgeon technique as well as pathologist experience and that some lymph nodes were too small to be found by surgeons and pathologists. The main problems were the method for staging these patients, the type of therapy to administer and the survival prognosis.

**Table 3:** Multivariate analyses of the overall survival in gastric cancer patients (Cox regression model, without the re-staged N system).

Variable	HR	95% CI	P value
OS in gastric cancer patients			
Tumour location	0.976	0.889–1.071	0.608
Tumour size	1.226	0.863–1.742	0.256
Borrmann type	1.103	0.943–1.289	0.220
Histological grade	1.148	0.999–1.320	0.051
T staging	1.501	1.285–1.753	<0.001
N staging	1.363	1.257–1.479	<0.001
Lymph nodes harvested	0.724	0.622–0.843	<0.001

Abbreviations: OS, overall survival; HR, hazard ratio; CI, confidence interval.

In our study, there were 346 patients with fewer than 15 lymph nodes harvested and 154 patients with more than 30 lymph nodes harvested. Some investigators have found that if the number of harvested lymph nodes is smaller, down-migration of the N stage may occur and conversely if the number is larger, up-migration of the N stage may occur [18]–[20]. We found that the prognosis of patients with 15–29 harvested lymph nodes was not significantly different from those with more than 30 nodes harvested in every N staging category (N0∶67.2 vs. 65.1 mo; N1∶65.0 vs. 61.5 mo; N2∶50.3 vs. 55.7 mo; N3a: 40.1 vs. 43.6 mo; N3b: 25.8 vs. 23.8 mo). We believe that the N staging would be accurate for a large number of harvested lymph nodes. Through our analysis, we found that the prognosis for patients with more than 30 lymph nodes harvested is better than those with fewer than 15 lymph nodes harvested, in every N stage. There was statistical significance for all N stages (N0∶59.7 vs. 66.6 mo, p = 0.027; N1∶60.5 vs. 63.5 mo, p<0.001; N2∶41.8 vs. 52.8 mo, p = 0.033; N3a: 30.9 vs. 41.5 mo; p = 0.028). There were down-migrations observed for the patients who had fewer than 15 lymph nodes harvested, which supports the AJCC’s recommendation that a minimum of 15 lymph nodes should be harvested for adequate staging.

**Table 4:** Multivariate analyses of the overall survival in gastric cancer patients (Cox regression model, including the re-staged N system).

Variable	HR	95% CI	P value
**OS in gastric cancer patients**			
Tumour location	0.992	0.904–1.089	0.868
Tumour size	1.218	0.858–1.730	0.270
Borrmann type	1.115	0.954–1.303	0.171
Histological grade	1.153	1.004–1.324	0.044
T staging	1.491	1.276–1.741	<0.001
N staging	0.696	0.525–0.924	0.012
Lymph nodes harvested	0.853	0.721–1.008	0.063
** Restaged N staging**	1.960	1.495–2.571	<0.001

Abbreviations: OS, overall survival; HR, hazard ratio; CI, confidence interval.

The other issue is how to stage gastric cancer patients with fewer than 15 lymph nodes harvested, especially patients with lymph node metastases. Empirical treatments from different oncologists make a substantial difference on the therapy and prognosis of those patients. The lymph node metastasis ratio is widely reported as an independent prognostic factor in gastric cancer [13], [14], [21]–[23]. However, different investigators use different cut-off values for the metastatic lymph node ratio to stage gastric cancer patients. For example, some investigators use 20% as a cut-off value while others use 50% as the cut-off value. Until now, there has been no uniform standard for the metastatic lymph node ratio cut-off values that would provide universal criteria for dividing the patients into different stage categories. The combination of the metastatic lymph node ratio and the AJCC’s TNM staging system has not yet been considered. In our trial, we divided those patients who had fewer than 15 lymph nodes harvested by the cut-off values defined by the AJCC’s 7^th^ TNM staging system as 0, 0.13 (2/15) and 0.40 (6/15). We then categorised those patients into the previous N staging group with the patients who had more than 15 lymph nodes harvested (0: N0; 0–0.13: N1; 0.13–0.40: N2; 0.40-: N3). Univariate and multivariate analyses were used to detect the effect of this staging method. We found our method has advantages over the previous N staging, and it should be considered as a method to reduce stage migration and to more accurately predict patient prognosis.

We found that the number of lymph nodes harvested was not an independent predictor. The reason may be that the predictive function of the lymph nodes harvested is covered by the re-categorised N staging in the multivariate analysis. If the re-categorised N staging is removed from the Cox’s regression analysis, the number of lymph nodes harvested is an independent prognostic factor. This also demonstrates the advantage of the re-categorised N staging system compared to the original N staging system. The function of the original N staging is preserved in the re-categorised N staging.

As a retrospective study, there were confounding factors that influenced the statistical analyses and conclusions. Lymph node metastasis is one of the most important prognostic factors for patients with gastric cancer. The staging of the disease may be improved by identifying micrometastases in the lymph nodes and identifying extranodal metastasis of the disease. Although our study showed that the re-categorised N staging system is more accurate than the traditional N staging system, further prospective studies would provide additional evidence supporting the use of our re-categorised N staging system and metastatic lymph node ratio as a standard for the N staging of gastric cancer.

### Conclusion

Harvesting a large number of lymph nodes (more than 30) in radical gastrectomy would not cause N-stage migration. Additionally, for those patients who had fewer than 15 lymph nodes harvested, accurate staging would best be accomplished by dividing by the metastatic lymph node ratio that is mentioned in the TNM staging system.
